# Effect of Ultrasonic Frequency Pulse Current on the Microstructure and Mechanical Properties of Ti6Al4V TIG Welded Joints

**DOI:** 10.3390/ma19020337

**Published:** 2026-01-14

**Authors:** Wanghui Xu, Xiaoyu Cai, Yu Li, Jing Wei, Chunlin Dong, Li Liu, Huan He

**Affiliations:** 1 School of Intelligent Manufacturing, Guangzhou Maritime University, Guangzhou 510725, China; wiggin93@163.com (J.W.); dchunlin@163.com (C.D.); liulimse@126.com (L.L.); 2 State Key Laboratory of Precision Welding & Joining of Materials and Structures, Harbin Institute of Technology, Harbin 150001, China; 3 College of Materials Science and Engineering, Beijing University of Technology, Beijing 100021, China; liyu123@emails.bjut.edu.cn; 4 College of Nuclear Equipment and Nuclear Engineering, Yantai University, Yantai 264005, China; welderhehuan@yahoo.com

**Keywords:** ultrasonic frequency pulse welding, TIG welding, Ti6Al4V, microstructure, mechanical properties

## Abstract

To enhance the performance of Ti6Al4V titanium alloy joints, ultrasonic frequency pulsed TIG welding was employed. The microstructure and mechanical properties of the joints were systematically investigated. Results show that the weld microstructure is predominantly composed of acicular α phase, lath α phase, and a minor amount of β phase. Compared with conventional TIG welding, the application of ultrasonic frequency pulse current effectively refined the grains, achieving an average grain size of 0.54 μm. Concurrently, the proportion of high-angle grain boundaries increased from 96.1% to 97.6%. The average hardness of the fusion zone exceeded that of the base metal and was significantly increased by the ultrasonic frequency pulse current, reaching 350 HV compared to 330 HV for conventional welds. Furthermore, the ultrasonic frequency pulsed TIG joints exhibited higher yield strength and elongation than their conventional welds. These findings demonstrate that introducing ultrasonic frequency current during TIG welding effectively improves the properties of Ti6Al4V welded joints.

## 1. Introduction

Titanium alloys are characterized by low density, high specific strength, excellent creep resistance, good weldability, and superior corrosion resistance [1,2,3,4]. These attributes have led to their extensive adoption in critical sectors such as aerospace, energy, chemical processing, and marine engineering [5,6]. The advancement of the aviation industry, in particular, creates a pressing demand for titanium alloy welded joints with enhanced mechanical performance, making research aimed at microstructural refinement and property improvement both timely and promising.

However, the Ti6Al4V alloy presents specific challenges: its physical properties lead to slow heat dissipation in the weld pool, and its chemical activity makes it prone to oxidation during welding [7,8]. Conventional arc TIG welding, characterized by relatively low energy density and a broad heat-affected zone, often exacerbates these issues. It can induce significant workpiece deformation and facilitate the formation of defects such as brittle phases, porosity, and cracks [9,10,11,12]. A major limitation of this process is its tendency to promote excessive grain coarsening, which directly compromises the mechanical integrity of the joint.

Consequently, developing methods to simultaneously elevate welding efficiency and joint quality in titanium alloys has emerged as a key research focus. Arc-ultrasonic TIG welding is a welding method in which ultrasonic frequency current is added to the welding current to modulate the thermal characteristics of the arc [13,14,15]. Some scholars found that for carbon steel, alloy steel, titanium alloy, composite materials and other materials, this welding method can refine the grain, degas and improve the mechanical properties of the joint. He et al. [16] found that arc-ultrasonic could refine weld grains and improve particle distribution. Zhu et al. [17] applied arc-ultrasonic to the TIG welding of MGH956 alloy, and the weld microstructure was significantly refined, the number of pores was reduced, and the weld plasticity was significantly improved. Lei et al. [18] found that arc-ultrasonic in 6061 aluminum alloy MIG welding can reduce the number of pores, increase the effective bearing area of the weld, and improve the strength of the fusion zone. Chen et al. [6] carried out Ti6Al4V titanium alloy arc ultrasonic TIG welding with an increase in ultrasonic frequency and excitation voltage; the weld grains become smaller and smaller, and the joint tensile strength also becomes higher and higher. Cao et al. [19] demonstrated that arc-ultrasonic can also be obtained in submerged arc welding of 09MnNiDR steel, and it helps to reduce the sensitivity of welded joints to welding heat input and improve the welding production efficiency. However, the influence mechanism of ultrasonic excitation current, especially on the microstructure and properties of joints, is not clear.

Previous studies have consistently demonstrated the beneficial effects of pulsed current on the microstructure and properties of titanium alloy welds. However, conventional pulsed TIG welding typically operates at low frequencies (around 100 Hz), which imposes a limit on its grain-refining capability. This limitation often results in the formation of coarse grains within the fusion zone, ultimately leading to a degradation of joint performance. To overcome this constraint, the present study elevates the pulse frequency to the ultrasonic range (25 kHz) for welding Ti6Al4V alloy. This shift from conventional thermal pulsation to ultrasonic frequency arc oscillation is postulated to exert a fundamentally different and more potent influence on the molten pool dynamics and solidification process. The high-frequency arc agitation is expected to induce significant grain refinement and other beneficial effects, thereby achieving a marked improvement in the mechanical properties of the welded joints.

In this paper, the ultrasonic frequency pulse TIG welding is used to weld Ti6Al4V titanium alloy, and the influence mechanism of ultrasonic frequency pulse current on the microstructure and properties of joints is systematically studied. The research results can provide a new method and theoretical basis for the development of Ti6Al4V titanium alloy structural parts in the field of aerospace, marine and chemical fields.

## 2. Materials and Methods

### 2.1. Ultrasonic Frequency Pulse TIG Welding System

**Figure 1:**
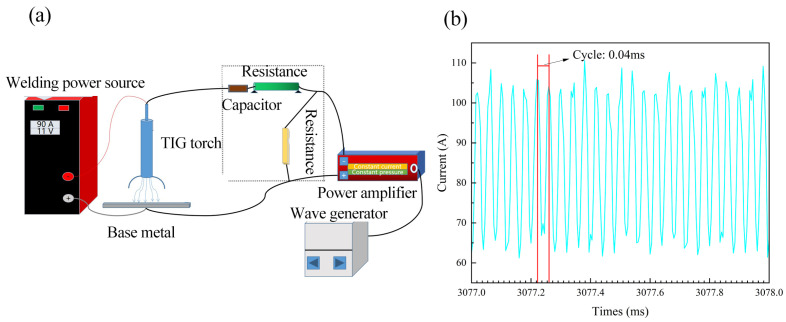
Ultrasonic f frequency pulse TIG welding system and arc current: (**a**), ultrasonic f frequency pulse TIG welding system; (**b**), arc current.

The ultrasonic frequency pulse TIG welding system primarily consists of an ultrasonic frequency pulse generation unit and a conventional TIG welding system, as illustrated in Figure 1. The pulse generation unit includes a waveform generator, a power amplifier, and an isolation coupler. The waveform generator can be configured to produce various voltage signal waveforms within a frequency range of 0–100 MHz. The power amplifier has an output frequency range of 10–100 kHz, an output current amplitude range of 0–50 A (peak), and a maximum power rating of 1750 W. The welding power source is an AC/DC TIG unit manufactured by EWM, with a maximum operating current of 520 A. To prevent the welding current from flowing into the ultrasonic pulse generation system, an isolation coupling device is installed at the output of the amplifier. This device allows the high-frequency current to pass while blocking the low-frequency welding current.

The welding procedure is as follows: the waveform generator produces an ultrasonic frequency pulse signal (set to 25 kHz in this study), which is then sent to the power amplifier. The amplifier receives this signal and outputs a corresponding 25 kHz current with an adjustable amplitude in the range of 0–50 A (peak). The output terminals of the power amplifier are connected in parallel to the positive and negative poles of the arc. Thus, the ultrasonic frequency pulse current and the welding current combine at the arc, collectively influencing the arc characteristics and the overall welding process.

Figure 1b shows the arc current waveform measured using a current sensor. The welding current was set to 90 A, and the superimposed ultrasonic frequency pulse current had a frequency of 25 kHz and an amplitude of 24 A. The results indicate that the arc current exhibits a period of 0.04 ms, corresponding to a frequency of 25 kHz, and fluctuates between approximately 63 A and 114 A. These values align with the theoretical expectation: arc current = welding current ± ultrasonic pulse current (90 + 24 = 114 A; 90 − 24 = 66 A). This confirms that the ultrasonic pulse current effectively modulates the arc and influences the welding process.

Furthermore, under the influence of the ultrasonic frequency pulse electric field, the arc undergoes periodic contraction at the same frequency. This causes the arc plasma to generate ultrasonic waves, which subsequently affect the molten pool and the solidification process. In other words, the arc serves not only as a heat source but also as an ultrasonic emission source.

### 2.2. Experimental Materials and Methods

The experimental material was a Ti-6Al-4V titanium alloy plate, its size was 200 mm × 100 mm × 10 mm, and its chemical composition is shown in Table 1.

**Figure 2:**
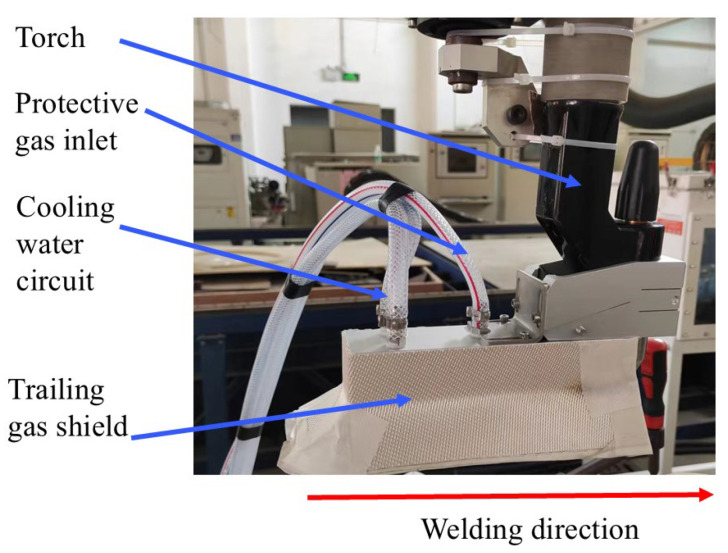
Photo of protective gas device.

Prior to welding, the workpiece surface was prepared by mechanical grinding to remove the oxide layer, followed by cleaning with ethanol. A DC TIG bead-on-plate welding was performed on a Ti-6Al-4V titanium alloy plate using an EWM TIG welding system (EWM GmbH, Altenkirchen, Germany). The welding parameters are shown in Table 2: The welding parameters were set as follows: current of 90 A, tungsten electrode diameter of 4 mm, travel speed of 150 mm/min, shielding gas of 99.999% pure argon, and gas flow rate of 15 L/min. To ensure adequate protection of the weld zone, the experimental setup was equipped with a trailing gas shield in addition to the primary torch, as illustrated in Figure 2. The supplemental gas flow rate was set at 10 L/min.

Under consistent welding conditions, ultrasonic frequency pulse currents of 6 A, 12 A, 18 A, 24 A, and 30 A were separately applied, with the frequency fixed at 25 kHz. 

After welding, cross-sectional specimens were taken perpendicular to the welding direction for metallographic and tensile testing. The metallographic samples were ground and polished, then etched using a solution with a volume ratio of HF:HNO_3_:H_2_O = 1:2:10. The microstructure of the welded joint was examined using a Zeiss Ario Imager M2m (Carl Zeiss AG, Oberkochen, Germany) digital metallographic microscope.

Grain size, orientation, and grain boundary distribution were analyzed using a Zeiss Gemini SEM 300 (Carl Zeiss AG, Oberkochen, Germany) scanning electron microscope, with an EBSD scanning step size of 0.12 μm. The fracture surfaces of the tensile specimens were also examined using the same SEM. Prior to EBSD analysis, the samples were electrophished in a mixture of 10 mL perchloric acid, 30 mL n-butanol, and 60 mL methanol. Electropolishing was conducted at –30 °C and 20 V for 1.5 min. During this process, the sample was held above a conductive spacer in a beaker filled with the electrolyte, ensuring no direct contact with the spacer. After electropolishing, the sample was promptly removed, rinsed with alcohol in an ultrasonic cleaner, and finally dried with air.

**Figure 3:**
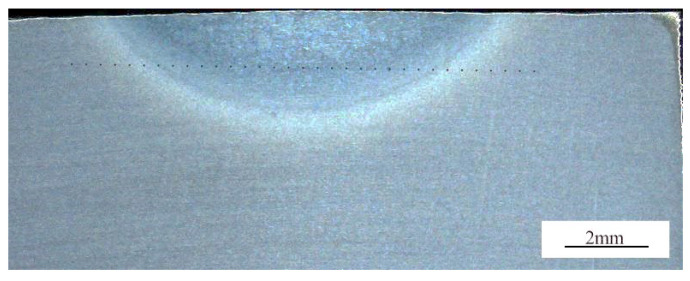
Hardness measurement position.

The microhardness of welded joints produced under different excitation currents was measured using a BUEHLER WILSON VH1202 microhardness tester (Buehler Ltd., Lake Bluff, IL, USA). Measurements were taken along the horizontal direction of the cross-section, as illustrated in Figure 3, with an interval of 0.25 mm between adjacent indentations. A test load of 1 kg and a dwell time of 10 s were applied for each measurement.

Tensile tests were performed on the specimens using an MTS CMT1505 universal testing machine (MTS Systems Corporation, Eden Prairie, MN, USA). Tensile testing of the welded joints was conducted in accordance with the Chinese national standard GB/T 2651-2023 (for tensile testing of welded joints) [20]. The yield strength (Rp0.2) was determined using an extensometer-aided offset method. A 634.11F-25 model extensometer (MTS Systems Corporation, Eden Prairie, MN, USA) was employed for strain measurement. The tensile specimens had an initial gauge length of 25 mm. The test was performed under a two-stage displacement control: a rate of 0.00025 mm/s was applied prior to yielding, followed by 0.0067 mm/s after yielding. 

## 3. Results

### 3.1. Effect of Ultrasonic Frequency Pulse Current on Weld Formation

**Figure 4:**
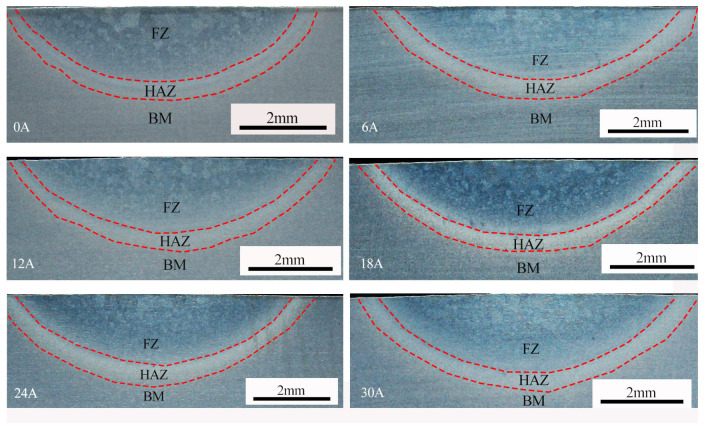
Cross-section of the weld with different ultrasonic frequency pulse currents.

As shown in Figure 4, sound titanium alloy joints can be achieved using the ultrasonic frequency pulse TIG welding method. Within the applied ultrasonic frequency pulse current range of 6 A to 30 A, the resulting welds are free from defects such as porosity.

**Figure 5:**
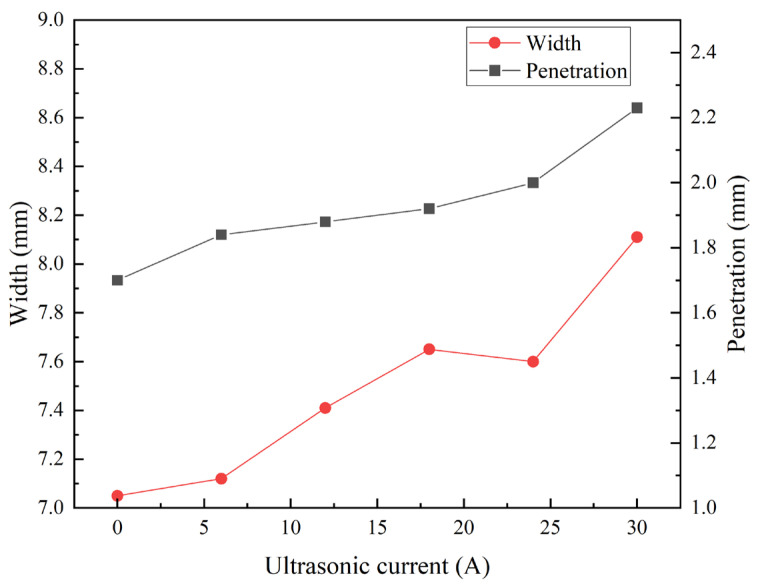
Weld width and penetration with different ultrasonic frequency pulse currents.

To evaluate the effect of the ultrasonic frequency pulsed current on weld geometry, this study measured the weld width, penetration depth, and depth-to-width ratio under different excitation parameters. As shown in Figure 5, conventional TIG welding produced a penetration of 1.7 mm, a weld width of 7.05 mm, and a corresponding depth-to-width ratio of 0.24. With the introduction of the ultrasonic frequency pulsed current, both weld width and penetration increased. For instance, at an ultrasonic frequency pulse current of 12 A, the weld penetration reached 1.84 mm, the width increased to 7.43 mm, and the depth-to-width ratio was 0.25. As the ultrasonic frequency pulse current was raised further, both penetration and width continued to grow. At the maximum applied current of 30 A, the penetration reached 2.23 mm, the weld width was 8.11 mm, and the depth-to-width ratio increased to 0.27.

In summary, compared to conventional TIG welds, the ultrasonic frequency current-assisted process produced a 31% greater penetration depth and a 15% wider bead, while the overall depth-to-width ratio remained largely unchanged. This significant change in geometry is attributed to the effect of ultrasonic excitation on the molten pool. The high-frequency agitation deforms and disrupts the stagnant and laminar boundary layers, generating turbulence that propagates into the bulk fluid. This perturbation reduces the thickness of the stagnant layer and disturbs the primary flow pattern, thereby enhancing convective heat transfer efficiency. Consequently, more heat is delivered to the central and lower regions of the weld, simultaneously promoting greater penetration and width.

### 3.2. Weld Microstructure

**Figure 6:**
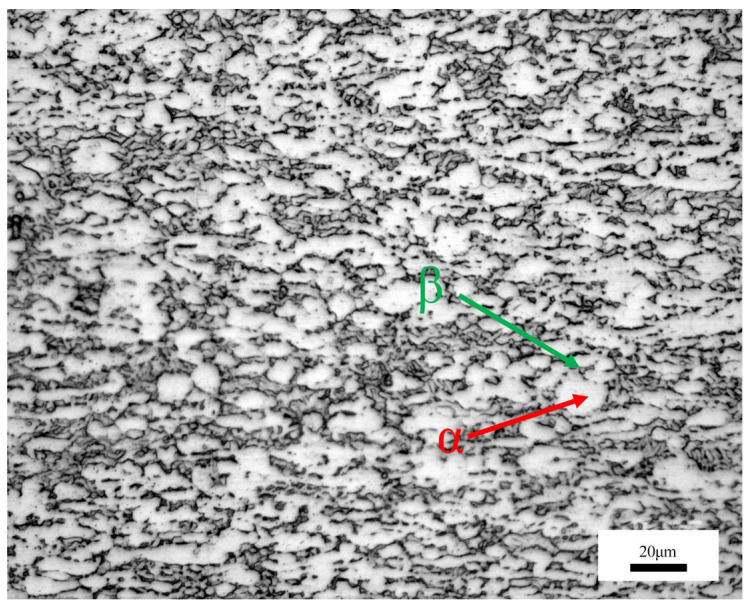
Microstructure of base metal.

Figure 6 illustrates the microstructure of the base metal, where the white and dark regions correspond to the α and β phases, respectively. As a result of the rolling process, the grains are aligned along the rolling direction. The Ti-6Al-4V titanium alloy, a typical α+β dual-phase alloy, is characterized by equiaxed α grains surrounded by the residual β phase at the grain boundaries.

**Figure 7:**
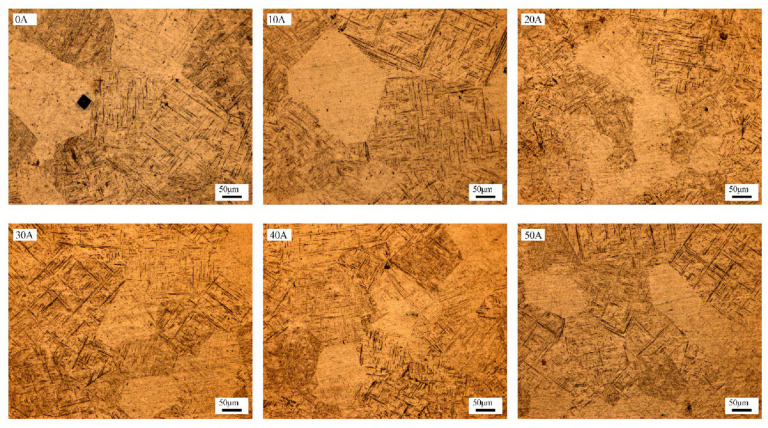
Microstructure under different ultrasonic frequency pulse currents.

The microstructure of the weld metal differs significantly from that of the base metal, having undergone a thermal cycle of heating, melting, solidification, and crystallization. During this process, the high-temperature β phase transforms into the α phase. As shown in Figure 7, the resulting weld microstructure consists of acicular martensite (α’) and acicular α phase. The nascent α phase laths are arranged in parallel, forming a basketweave morphology. Compared with conventional TIG welding (represented by the 0 A condition in Figure 7), the ultrasonic frequency pulse TIG process produces a markedly refined grain structure and a denser basketweave architecture.

### 3.3. Weld Grain Size and Grain Boundary

**Figure 8:**
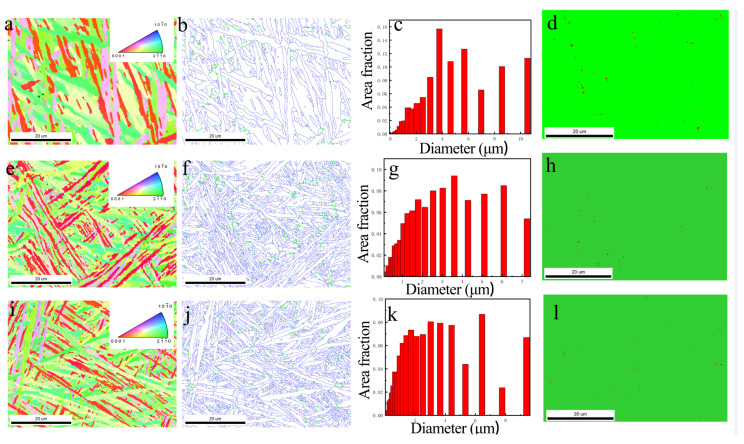
IPF maps, grain boundary maps, grain size distribution maps and phase proportion distribution maps of different ultrasonic frequency pulse currents: (**a**–**d**), conventional TIG; (**e**–**h**), ultrasonic frequency pulse current 6 A; (**i**–**l**), ultrasonic frequency pulse current 30 A.

To further investigate the influence of ultrasonic frequency pulse TIG welding on the metallographic structure, three weld samples produced with ultrasonic frequency pulse currents of 0 A, 6 A, and 30 A were selected for electron backscatter diffraction (EBSD) analysis. Their grain size and grain boundary characteristics were examined, as shown in Figure 8.

Figure 8a,e,i present the inverse pole figure (IPF) maps, where crystal orientations are represented by a color spectrum. All three samples exhibit a fully acicular microstructure, consistent with the optical micrographs. The orientations of these acicular structures appear relatively random. Figure 8b,f,j show the grain boundary distributions, with boundaries between 2° and 15° defined as low-angle grain boundaries (green) and those between 15° and 180° as high-angle grain boundaries (blue). In the conventional TIG weld (0 A), the fraction of high-angle grain boundaries is 0.96. This value increases to 0.97 at 6 A and further to 0.98 at 30 A, indicating that the proportion of high-angle grain boundaries rises with increasing ultrasonic frequency pulse current.

Figure 8c,g,k display the grain diameter distributions. With the application of ultrasonic frequency pulse current, the proportion of larger grains decreases, while that of finer grains increases. The measured average grain sizes for the three samples are 0.86 μm, 0.58 μm, and 0.54 μm, respectively. These results confirm that ultrasonic frequency pulse current effectively refines the weld grains, and the degree of refinement becomes more pronounced as the ultrasonic frequency pulse current increases.

Figure 8d,h,l illustrate the phase distribution, in which the green and red regions represent the α and β phases, respectively. It can be observed that the conventional TIG weld contains a relatively high fraction of β phase. With the introduction of the ultrasonic frequency pulse current, the proportion of the β phase decreases, and this effect is enhanced at higher ultrasonic frequency pulse currents.

### 3.4. Microhardness of Welded Joints

**Figure 9:**
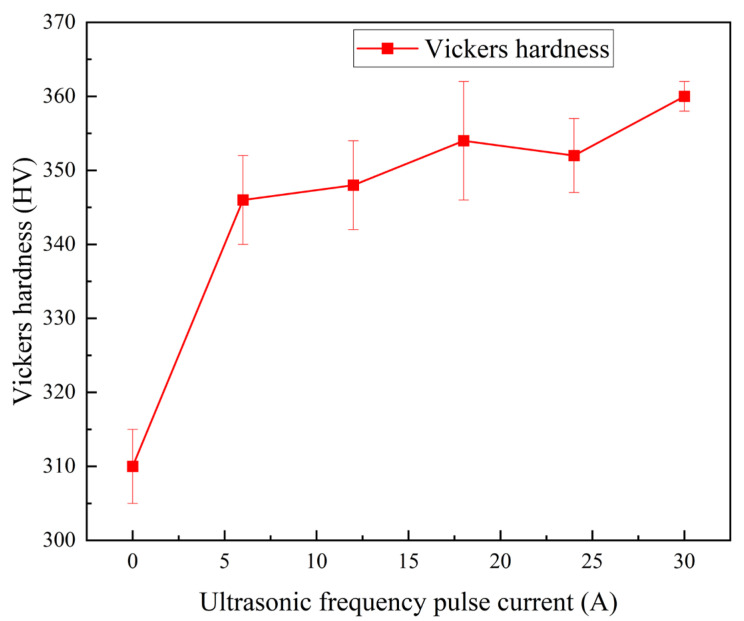
Average weld microhardness at different ultrasonic frequency pulse currents.

Figure 9 shows the average microhardness measured in the fusion zone under different ultrasonic frequency pulse currents. The fusion zone of conventional TIG welds exhibits an average hardness of approximately 334 HV. When an ultrasonic frequency pulse current of 6 A is applied, the average hardness increases by about 12 HV, reaching 346 HV. A further rise in hardness is observed at 30 A, where the average microhardness attains around 360 HV. These results indicate that the introduction of ultrasonic excitation effectively strengthens the fusion zone, with the extent of hardening being positively correlated with the magnitude of the applied ultrasonic frequency pulse current.

### 3.5. Tensile Strength and Fracture Analysis

**Figure 10:**
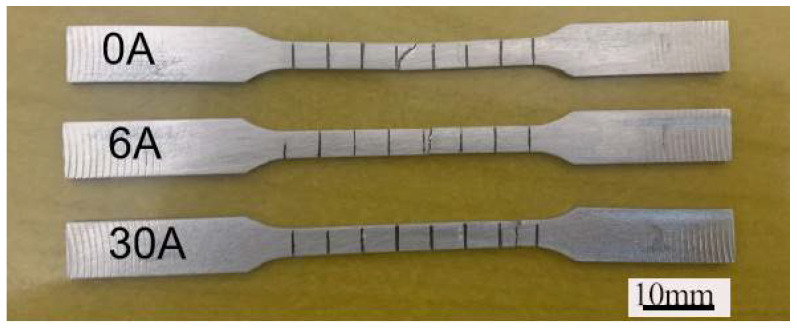
Macroscopic fracture locations of welded joints under different excitation currents.

Figure 10 shows the macroscopic fracture locations of welded joints produced with different ultrasonic frequency pulse currents. In conventional TIG-welded joints, tensile fracture occurs within the weld metal. The fracture surface is aligned parallel to the maximum shear stress and at approximately 45° to the tensile direction, indicating a micromechanical failure mode dominated by microvoid coalescence. At an ultrasonic frequency pulse current of 6 A, a fracture still takes place in the weld zone. However, when the ultrasonic frequency pulse current is increased to 30 A, the fracture location shifts to the interface between the heat-affected zone and the base metal.

**Figure 11:**
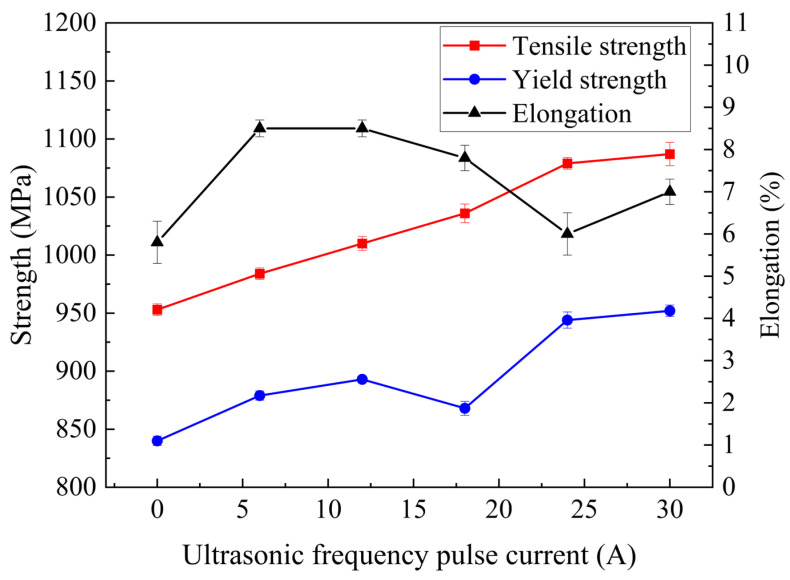
Tensile strength and yield strength of welded joints under different ultrasonic frequency pulse currents. Tensile strength, yield strength and elongation of welded joints under different ultrasonic frequency pulse currents.

**Figure 12:**
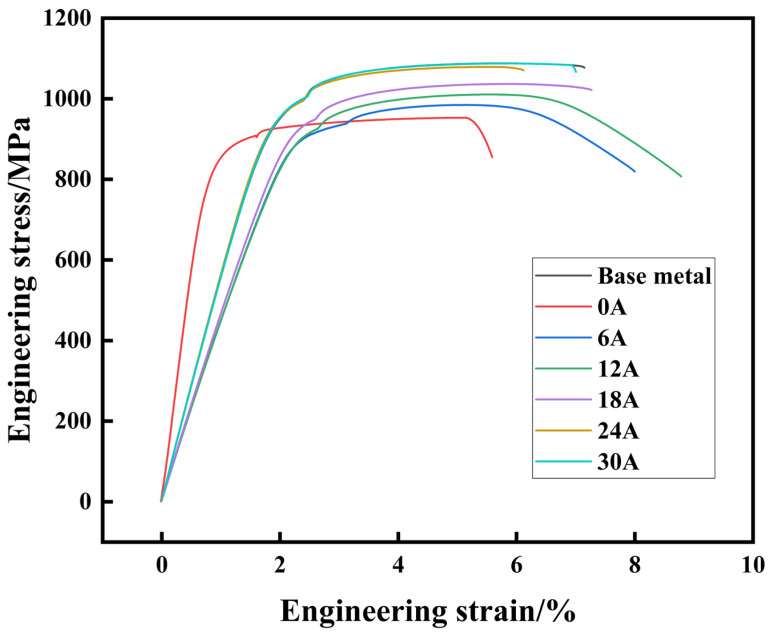
Tensile strength and yield strength of welded joints under different ultrasonic frequency pulse currents. Engineering stress-strain curves of welded joints under different ultrasonic frequency pulse currents.

Figure 11 summarizes the tensile strength and yield strength results. The yield strength was determined using the extensometer-aided offset method (Rp0.2). Figure 12 presents the corresponding stress–strain curves obtained from the tensile tests. The base metal exhibited a tensile strength of 1078 MPa, a yield strength of 954 MPa, and an elongation of 7.5%. The conventional TIG joint exhibits a tensile strength of 957 MPa and a yield strength of 848 MPa. With the application of a 30 A ultrasonic current, the tensile strength increases to 1087 MPa and the yield strength rises to 952 MPa. The tensile and yield strengths were increased by 13.3% and 12.3%, respectively. As illustrated in Figure 11, the yield strength shows a clear increasing trend with higher ultrasonic current. The elongation of the joints was also measured during tensile testing. The conventional TIG joint shows an elongation of 5.8%, which increases to 8.5%, representing a 46.6% improvement relative to the conventional TIG case. These results demonstrate that both the strength and ductility of the welded joint are enhanced with increasing ultrasonic frequency pulse current.

**Figure 13:**
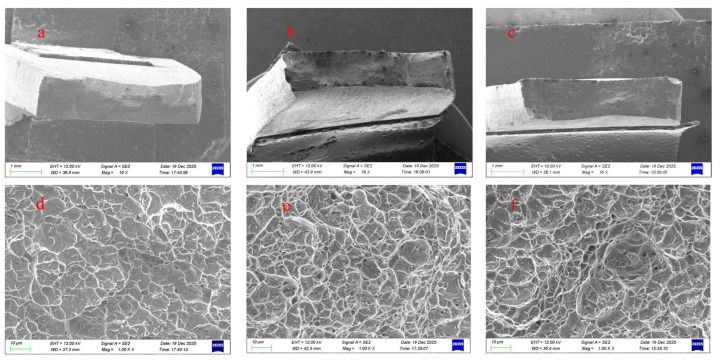
SEM images of tensile fracture surfaces under different ultrasonic frequency pulse currents: (**a**) 0 A, low magnification; (**b**) 6 A, low magnification; (**c**) 30 A, low magnification; (**d**) 0 A, high magnification; (**e**) 6 A, high magnification; (**f**) 30 A, high magnification.

Figure 13 presents SEM images of the tensile fracture surfaces. Figure 13a,d show the fracture morphology of the conventional TIG weld, which exhibits a river pattern characteristic of brittle transgranular cleavage with no visible dimples. The absence of significant plastic deformation, combined with prior microstructural observations, suggests that the weld fails in a completely brittle manner, which is attributed to its coarse-grained structure. With the introduction of ultrasonic current (Figure 13e,f), the number of dimples on the fracture surface increases noticeably. At 30 A (Figure 13c,f), the dimples become larger, deeper, and more uniformly distributed, indicating a significant improvement in weld plasticity.

## 4. Discussion

### 4.1. Influence of Ultrasonic Frequency Pulse Current on Weld Dimensions

As shown in Figure 4, ultrasonic frequency pulse TIG welding yields greater penetration depth and weld width compared with conventional TIG welding. Both dimensions exhibit an increasing trend with higher ultrasonic pulse current. This behavior can be attributed to two main factors: the arc constriction effect induced by the ultrasonic frequency pulse current, and the associated increase in effective heat input [20].

**Figure 14:**
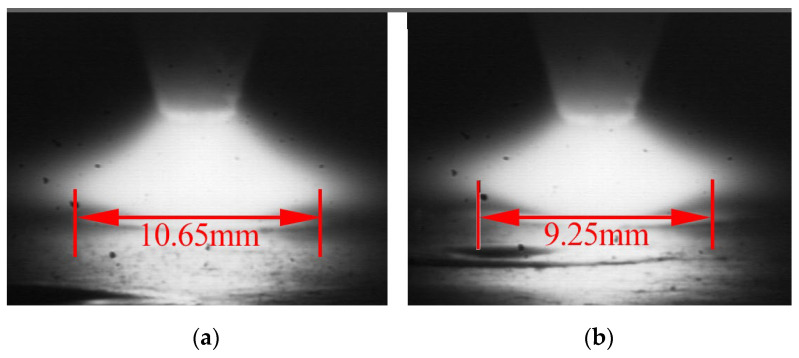
Arc morphology under different ultrasonic frequency pulse current: (**a**) conventional TIG; (**b**) ultrasonic frequency pulse current 30 A.

To verify whether the ultrasonic excitation influences the arc profile, arc images were captured under conventional TIG and 30 A ultrasonic current conditions, as shown in Figure 14. The maximum arc width measured in conventional TIG welding was 10.65 mm, while under ultrasonic frequency pulse current, it decreased to 9.25 mm. This confirms that the arc contracts under the influence of the ultrasonic frequency pulse current, leading to increased arc pressure and thereby promoting deeper weld penetration. 

The superimposed ultrasonic frequency current takes the form of a sinusoidal AC waveform. Its net average current is zero; however, the resulting arc heating is dictated by the root-mean-square (RMS) effective current. Formula (1) provides the fundamental relationship between this effective current value and the peak amplitude of the AC pulse.

(1) $$I_{rms}=\sqrt{\frac{1}{T}\int_{0}^{T}{i(t)}^{2}dt}=\sqrt{\frac{1}{T}\int_{0}^{T}{I_{m}}^{2}{sin}^{2}(wt)dt}=\frac{I_{m}}{\sqrt{2}}$$

In this context, *I_rms_* denotes the root-mean-square (RMS) or effective current of the AC waveform, *I_m_* is its peak current, and *T* represents the period of the ultrasonic frequency. The resulting Joule heat is governed by the formula:

(2) $$Q_{rms}={I_{rms}}^{2}R$$

where *Q_rms_* is the heat generated in the resistor and *R* represents the equivalent arc resistance.

Therefore, although the superposition of a high-frequency AC current onto the DC welding current does not increase the average current, it elevates the effective RMS current. Consequently, the total thermal input to the weld pool increases. This provides the theoretical explanation for the observed increase in weld width with increasing peak ultrasonic current: higher peak current leads to a higher *I_rms_* and thus greater heat generation.

In summary, the enhancement in both penetration and width can be attributed to the combined effects of (1) the electromagnetic force-induced arc constriction and (2) the increased thermal input resulting from the rise in effective current under ultrasonic excitation.

### 4.2. Effect of Ultrasonic Frequency Pulse Current on Microstructure

EBSD results indicate that the introduction of the ultrasonic arc significantly refines the grain structure. The average grain size decreases from 0.86 μm in conventional TIG welds to 0.54 μm with ultrasonic excitation, exhibiting a clear decreasing trend as the ultrasonic current increases. This grain refinement is primarily attributed to the stirring effect induced by the ultrasonic frequency pulse current [21].

**Figure 15:**
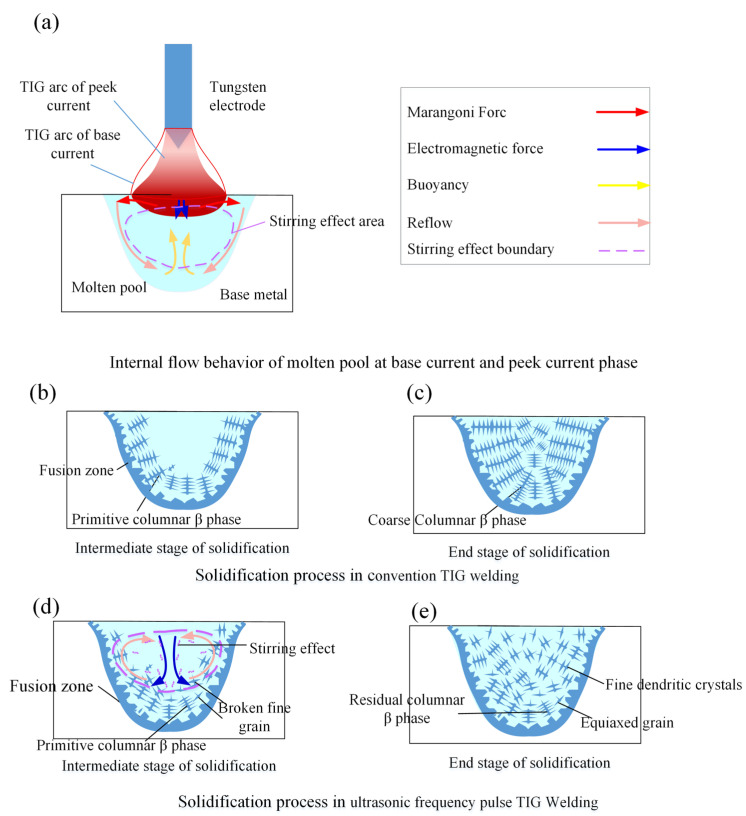
Schematic diagram of the influence of ultrasonic frequency pulse current on the solidification process of the molten pool. Schematic diagram of the influence of ultrasonic frequency pulse current on the solidification process of the molten pool: (**a**), internal flow behavior of molten pool at base current and peek current phase; (**b**), the solidification intermediate stage in conventional TIG welding; (**c**), the solidification end stage in conventional TIG welding; (**d**), the solidification intermediate stage in ultrasonic frequency pulse TIG welding; (**e**), the solidification end stage in ultrasonic frequency pulse TIG welding.

As illustrated in Figure 15, the forces exerted by the arc on the molten pool primarily include surface tension, electromagnetic force, plasma drag force, and buoyancy. For titanium alloys, the surface tension exhibits a negative temperature coefficient; consequently, the resulting Marangoni flow is directed from the hotter arc center towards the cooler periphery. In contrast, both the electromagnetic force and the plasma drag force, which are strongly dependent on the welding current and arc configuration, are predominantly oriented downward toward the pool bottom. Buoyancy, arising from density variations due to temperature gradients, generally acts upward.

Figure 15a schematically depicts the arc morphology and molten pool flow during the base and peak stages of the ultrasonic frequency pulsed current. During the base stage (lower current), the electromagnetic and plasma drag forces are minimal. The pool surface remains relatively flat, and fluid flow is dominated by surface-tension-driven convection and thermal buoyancy, circulating from the surface center outward and then back along the solidification front. During the peak stage, the increased current intensifies the arc forces, notably depressing the pool center. This creates a strong downward flow along the central axis, with return flow along the sidewalls, thereby compressing the surface-tension-driven flow to the periphery and establishing a fundamentally altered flow field.

Furthermore, the ultrasonic frequency modulation introduces additional effects such as the skin effect and enhanced electromagnetic agitation. These effects significantly amplify the arc forces. The resultant high-frequency oscillation and rapid flow-field alterations create a vigorous stirring zone in the central region of the pool, which is the primary factor responsible for grain refinement.

For comparison, the solidification process in conventional TIG welding (i.e., with zero ultrasonic frequency current) is shown in Figure 15b,c. Solidification initiates at regions of high thermal conductivity, leading to the formation and growth of columnar grains until the entire weld solidifies. The resulting microstructure is thus dominated by coarse columnar prior-β grains.

In contrast, with the application of ultrasonic frequency current, the periodic stirring within the molten pool (Figure 15d) causes fragmentation of the dendritic arms. These fragments act as additional nucleation sites, increasing the nucleation density and effectively refining the coarse columnar structure. Although grain growth continues during subsequent solidification (Figure 15e), the final microstructure is significantly refined compared to that of conventional TIG welds.

### 4.3. Influence of Ultrasonic Frequency Pulse Current on Mechanical Properties

Tensile test results demonstrate that the tensile strength, yield strength, and elongation of the weld are all improved with the application of the ultrasonic frequency pulse current. According to the EBSD analysis, the ultrasonic excitation produces significant grain refinement, which directly enhances the mechanical properties of the material. This improvement occurs because the refined microstructure, consisting predominantly of fine equiaxed grains, strongly impedes dislocation glide and restricts the movement of slip systems. This strengthening mechanism leads to the observed simultaneous increase in both strength and ductility.

The relationship between grain size and yield strength is well described by the Hall-Petch equation [22]:

(3) $$\sigma_{s}=\sigma_{0}+\frac{k}{\sqrt{d}}$$

where *σₛ* is the yield strength, *σ_0_* and *k* are material constants, and *d* is the average grain diameter. This equation establishes an inverse relationship between grain size and yield strength: finer grain structures result in higher yield strength.

During welding, the arc melts the base metal. As the arc moves away, the molten pool cools and initiates solidification. The application of ultrasonic frequency current actively interferes with this crystallization process, promoting grain refinement and, consequently, enhancing the mechanical properties of the weld. Although the welding process also creates a heat-affected zone (HAZ) where grain coarsening can occur and degrade properties, the influence of the ultrasonic current is predominantly confined to the molten weld metal due to the lack of fluid flow in the solid-state HAZ. Therefore, the grain refinement discussed in this study pertains specifically to the fusion zone. The underlying strengthening mechanism is elucidated by the Hall-Petch relationship, which establishes that a finer grain structure increases the total grain boundary area per unit volume. These boundaries act as potent barriers to dislocation motion, thereby elevating the material’s yield strength.

In the present experiments, the superposition of ultrasonic frequency pulsed current induces high-frequency variations in arc forces, resulting in forced oscillations within the molten pool. This creates intense fluid stirring, particularly in the central region. The vigorous agitation fragments developing dendrites, effectively refining the grain structure. According to the Hall–Petch principle, this refinement directly contributes to higher yield strength. Furthermore, as the ultrasonic current amplitude increases, the stirring intensifies, producing even finer grains, which accounts for the observed overall upward trend in yield strength.

## 5. Conclusions

Microstructure and mechanical properties of TIG-welded Ti-6Al-4V titanium alloy joints were investigated, leading to the following conclusions:

(1) The ultrasonic frequency pulse TIG welding process produces well-formed titanium alloy welds. Both weld width and penetration depth increase with higher ultrasonic frequency pulse current.
(2) Compared with conventional TIG welding, the ultrasonic frequency pulse TIG process results in progressive grain refinement and increased equiaxed grain formation as the ultrasonic current increases. The proportion of high-angle grain boundaries and the α-phase fraction both rise with increasing ultrasonic current. The average grain size decreases by 37%, from 0.86 μm in conventional TIG welding to 0.54 μm with ultrasonic assistance.
(3) The microhardness of the fusion zone increases from 334 HV to 360 HV with ultrasonic application, primarily due to grain refinement. Both yield strength and elongation improve with higher ultrasonic currents. At the ultrasonic current of 30 A, the joint achieves a yield strength of 954 MPa, a tensile strength of 1078 MPa, and an elongation of 7.5%. The tensile and yield strengths increased by 13.3% and 12.3%, respectively.
(4) The ultrasonic frequency pulse current causes arc contraction, increasing arc pressure and enhancing molten pool stirring. This effect disrupts columnar grain growth, promotes grain refinement, and ultimately improves the mechanical strength of the weld.

## Figures and Tables

**Table 1:** Chemical composition of TC4 titanium alloy (wt%).

	H	C	N	O	Fe	V	AI	Ti
TC4	0.0014	0.013	0.014	0.15	0.30	3.92	6.06	Bal.

**Table 2:** Welding parameters.

Welding Current/A	Torch–Workpiece Distance/mm	Travel Speed/mm·min^−1^	Electrode Diameter/mm	Gas Flow Rate/L·min^−1^	Ultrasonic Frequency Pulse Current/A	Frequency/kHz
90	4	150	4	15	0, 6, 12, 18, 24, 30	25

## Data Availability

The original contributions presented in this study are included in the article. Further inquiries can be directed to the corresponding author.
